# The Influence of COVID-19 Lockdown on Physical Activity, Sedentary Behavior and Social Support Specific to Physical Activity Among Belgian Adults

**DOI:** 10.3389/fspor.2021.716386

**Published:** 2021-09-20

**Authors:** Pierre Van Luchene, Fanny Detemmerman, Cécile Delens

**Affiliations:** Faculty of Motor Sciences, Institute for the Analysis of Change in Contemporary and Historical Societies, Université Catholique de Louvain, Louvain-la-Neuve, Belgium

**Keywords:** active behavior, sedentary behavior, psychosocial determinants, COVID-19, life-change event

## Abstract

In Belgium, lockdown measures were taken to counter the spread of COVID-19. This major life-change event may disrupt a person's daily routine and influence health behaviors. Although measures were restrictive, citizens were encouraged to engage in physical activity (PA) behavior in order to maintain well-being. Social support specific to PA (SSPA) had been highlighted as an important psychosocial factor in initiating and/or maintaining active behavior. The main aims of this study were to explore the influence of COVID-19 lockdown on PA and sedentary behavior, and on SSPA from family and from friends; and investigate the potential differences in terms of professional status. An online survey was distributed during the 1st weeks of the lockdown. A total of 272 Belgian adults responded to the survey. The findings show no significant difference between prior to and during lockdown with regard to the amount of PA. The results show a significant increase in sedentary behavior among the entire sample, workers and retirees. The findings also suggest that the support of other individuals is particularly useful for certain population groups such as retirees. Given the importance of the levels of PA and sedentariness as health behaviors preceding a major life-change event such as a lockdown, there is a need to promote these health behaviors during normal life in order for the population to remain active throughout their lifespan.

## Introduction

Life experiences that may greatly influence an individual's daily routine are referred to as life-change events (Holmes and Rahe, 1967) and have been defined as significant experiences involving a relatively abrupt change that may produce profound and long-lasting effects (Olafsson and Svensson, 1986; Settersten and Mayer, 1997). Stressful events may influence health behaviors either by disrupting an individual's ability to engage in such behaviors, or by increasing engagement in negative health-related behaviors. Life-change events may create emotional distress and disrupt a person's daily routine, thus affecting the commitment to be physically active (Engberg et al., 2012). A systematic review focused on the influence of life-change events on physical activity (PA) identified 5 life-event domains: changes in employment status, changes in residence, changes in physical status, changes in relationships, and changes in family structure (Allender et al., 2008). In a longitudinal study among young adults, Paluch et al. (2018) reported that life-change events can result in both positive and negative changes in PA behaviors.

On 11th March 2020, the World Health Organization declared the coronavirus disease 2019 (COVID-19) to be a global pandemic. In order to deal with the spread of COVID-19, many public health recommendations and regional and national government measures have resulted in numerous restrictions on daily living including social distancing, isolation and home lockdown. Worldwide, this COVID-19 lockdown had a negative effect on PA, and increased sedentary behavior (Ammar et al., 2020; Tison et al., 2020). Nevertheless, individuals were able to adapt quickly in the face of lockdown to improve their health behaviors, such as physical activity (López-Bueno et al., 2020). In Belgium, the government opted for the first lockdown measures starting on 13th March 2020, whereby schools were closed, homeworking became the new standard whenever possible, and social relations were greatly restricted. Based on these measures, lockdown can impact different areas of life by changing people's working conditions, their relationships, or their health status. However, although sports centers, gyms and other indoor sports facilities were closed, citizens were encouraged to engage in PA behavior in order to maintain well-being. More specifically, citizens were encouraged to engage in PA at home, or outdoors, alone, with members of the same household, or with a single friend.

Many behavior change theories (Bandura, 1986; Rosenstock et al., 1988), as well as health behavior adoption theories (Prochaska and DiClemente, 1983; Ajzen, 1991; Schwarzer, 2008) have highlighted the importance of psychosocial factors in the form of social support (SS) in initiating and/or maintaining active behavior. Social support was defined as any behavior that assists a person in achieving a desired goal (Taylor et al., 1994). This support for the individual can come from several sources within that individual's social network (e.g., family, friends, co-workers). The composition of this individual's social network seems to be influenced by life-change events, especially those related to education and employment. Indeed, starting higher education at college or University can be accompanied by significant changes in terms of residence, distance from family, and the creation of new social contacts. The world of work offers opportunities to create and maintain social networks. At the time of retirement, contact with colleagues may decrease, while this life-change event increases the time available to be with family, friends, or neighbors. In a cohort study focusing on the evolution of the social network during the transition to retirement, Kauppi et al. (2021) suggested that the reduction in social relationships is more likely to be associated with retirement than with the individual's own aging. The SS specific to PA (SSPA) can take several forms, such as emotional support, informational support, instrumental support, modeling, and co-participation (Duncan et al., 2005; Laird et al., 2016). There have been a number of systematic reviews of the literature on the influence of SSPA as a determinant of PA (Lindsay Smith et al., 2017; Scarapicchia et al., 2017; Van Luchene and Delens, 2021). With regard to college and University students (Van Luchene and Delens, 2021), there appears to be a positive association between SSPA from friends and family and PA. According to a systematic review of healthy adults (Scarapicchia et al., 2017), there is a small positive association between support for PA on the part of friends, and future PA. With regard to older adults, higher amounts of SSPA from all sources combined, and from family particularly, are associated with higher levels of PA, or meeting PA guidelines (Lindsay Smith et al., 2017).

As the entire population was affected by the lockdown at the same time and given the relational and professional drastic changes linked to COVID-19 lockdown, understanding the evolution of SSPA from friends and family during this unprecedented life-change event, and its potential link with changes in PA and sedentary behaviors is scientifically interesting in order to better understand reactions to life-change events with regard to PA practices. Therefore, the aims of this study were to: (1) explore the perception and influence of COVID-19 lockdown on PA and sedentary behaviors, and the potential differences in terms of professional status; (2) explore the influence of COVID-19 lockdown on SSPA from family and from friends, and the potential differences in terms of professional status; and (3) explore the potential links between changes in SSPA from family and from friends and changes in PA and sedentary behaviors, and the potential differences in terms of professional status.

## Materials and Methods

### Participants and Procedure

An online survey in French was disseminated from the authors' social networks and by a publication on the official site of the Catholic University of Louvain. Convenience sampling was used to select the participants of the study. For this study, only a part of the entire online survey was used. The survey was carried out between April 1st and April 22nd, 2020, corresponding to the first moments of lockdown in Belgium in order to best analyze the lockdown as a life-change event disrupting the daily life of individuals. Two inclusion criteria in terms of age and place during the COVID-19 lockdown were employed. With regard to age, participants had to be over the age of 18 in order to allow the authors to compare different professional statuses (students, workers, and retirees). With regard to place during the COVID-19 lockdown, because the lockdown measures applied were specific to each country, participation was limited to Belgian adults. The study sample consisted of 272 participants with a mean age 42.6 (SD = 16.4) years who completed the online survey concerning the following dimensions: demographic information, perceived influence of lockdown, physical activity, sedentary behavior, social support specific to physical activity from family and social support specific to physical activity from friends.

### Measures

#### Demographic Information

Demographic questions consisted of 5 items. Items included age, gender, level of education, professional status, and parenthood status. Demographic information pertinent to this study were age, gender, and professional status.

#### Perceived Influence of Lockdown

The perceived influence of lockdown on the participants' PA was assessed using a single question “Specify the overall influence of COVID-19 lockdown on your physical activities practice.” Participants were asked to select one of three levels of perceived influence: incentive to engage in PA, no influence on PA, or barrier to engage in PA.

#### Physical Activity and Sedentary Behavior

The level of exercise prior to lockdown was assessed using the Stage of Change to Exercise Behavior Scale (SOC) proposed by Marcus et al. (1992). The SOC asks participants to select one of five ordered statements that best describes the level of exercise prior to lockdown: precontemplation, contemplation, preparation, action, and maintenance. A modified version of the International Physical Activity Questionnaire short form (IPAQ-SF) (Craig et al., 2003) was used twice to assess the participants' PA and sedentary behavior for 7 consecutive days in a typical week prior to lockdown, and during the last 7 days of lockdown. Participants were asked to recall the amount of time they spent doing moderate to vigorous, light, sitting and lying, or sleeping activities.

#### Social Support Specific to Physical Activity

The SSPA was measured using the Social Support for Exercise Scale (SSES) (Sallis et al., 1987). The SSES consists of a 15-item scale with regard to family support and 5-item scale with regard to friends support, that assesses the frequency of such support over the 3 months prior to lockdown and since the beginning of lockdown using a 5-point scale (1 = none to 5 = very often). The current study found the SSES scores to have a good internal consistency (SSPA from family prior to lockdown: α = 0.84; SSPA from family during lockdown: α = 0.86; SSPA from friends prior to lockdown: α = 0.80; SSPA from friends during lockdown: α = 0.83).

#### Calculated Data

The evolution of PA, sedentariness, SSPA from family, and SSPA from friends, between prior to and during lockdown, were computed by subtracting the values prior to lockdown from the values during lockdown (evolution = during lockdown—prior to lockdown). Positive data means an increase between prior to and during lockdown and negative data means a decrease.

#### Data Analyses

Data were imported into Statistical Package for Social Sciences software version 25 (IBM Corp, Armonk, NY, USA) in order for it to be analyzed. When outliers were found, they were recoded as missing values for the variable in question. Preliminary analyses were conducted which included checking for missing data, removing multivariate outliers, and running descriptive statistics such as means, standard deviations, reliabilities, and normality. Non-parametric statistics were conducted because normality was not respected in at least one of the professional status subgroups for each variable investigated. After descriptive analyses, correlation analyses were conducted to gain a preliminary insight regarding the relationships among the predictor and outcome variables. Then, the Wilcoxon test, the Kruskal-Wallis test, and the Mann-Whitney test were conducted to determine whether the means of the variables significantly differed between professional statuses. Finally, general linear models were carried out according to professional status. The alpha level of 0.05 was used for all statistical analyses.

## Results

### Descriptive Statistics, Evolutions in PA, Sedentary Behavior and SSPA Over Time, and Comparisons in Terms of Professional Status

A total of 272 participants (76.47% female) between 18 and 76 years of age filled out the online survey: 38 were college or university students (71.05% female; 63% in maintaining stage), 195 were workers (77.95% female; 63% in maintaining stage), and 39 were retirees (74.36% female; 67% in maintaining stage). Table 1 provides an overview of the participant characteristics.

**Table 1:** Participant characteristics.

**Professional status**	**N (%)**	**Mean age (SD)**	**Gender (%)**	**Stage of change (%)**
Students	38 (13.97)	22.87 (4.98)	M: 10 (26.32) F: 27 (71.05) X: 1 (2.63)	S1: 5 (13.16) S2: 4 (10.53) S3: 3 (7.89) S4: 2 (5.26) S5: 24 (63.16)
Workers	195 (71.69)	41.65 (12.91)	M: 42 (21.54) F: 152 (77.95) X: 1 (0.51)	S1: 22 (11.28) S2: 13 (6.67) S3: 17 (8.72) S4: 20 (10.26) S5: 123 (63.08)
Retirees	39 (14.34)	67.59 (5.13)	M: 10 (25.64) F: 29 (74.36)	S1: 10 (25.64) S2: 2 (5.13) S3: 1 (2.56) S4: 0 (0.00) S5: 26 (66.67)
Total	272 (100)	42.57 (16.4)	M: 62 (22.79) F: 208 (76.47) X: 2 (0.74)	S1: 37 (13.60) S2: 19 (6.98) S3: 21 (7.72) S4: 22 (8.09) S5: 173 (63.60)

*M, male; F, female; X, other*.

*Stage of change: S1, precontemplation stage; S2, contemplation stage; S3, preparation stage; S4, action stage; S5, maintaining stage*.

**Figure 1:**
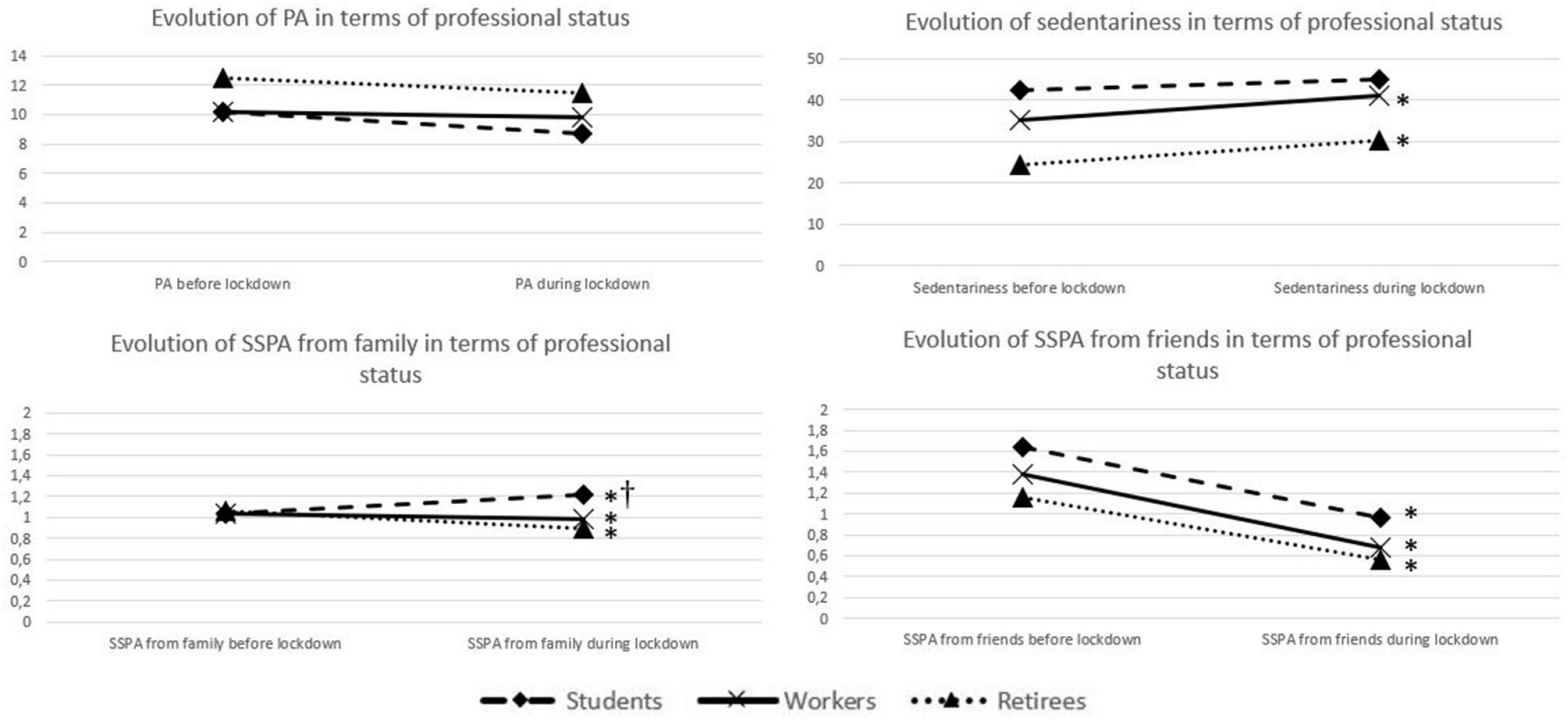
Evolutions over time and comparisons in terms of professional statuses of PA, sedentariness, SSPA from family and SSPA from friends. *indicates significant differences between prior to and during lockdown (*P* < 0.05). ^†^indicates significant difference between the evolution of students and the evolution of workers and retirees (*P* < 0.05).

Means and standard deviations for the variables of PA, sedentariness, SSPA from family and SSPA from friends are computed for the entire sample and by professional status (see Table 2). Statistical analyses show no significant difference between prior to and during lockdown with regard to the amount of PA among the entire sample, and in terms of professional status. There are significant increases in sedentariness among the entire sample (*p* = 0.000), workers (*p* = 0.000) and retirees (*p* = 0.000); a significant increase in SSPA from family among students (*p* = 0.028); significant decreases in SSPA from family among the entire sample (*p* = 0.050), workers (*p* = 0.019) and retirees (*p* = 0.026); and significant decreases in SSPA from friends among the entire sample (*p* = 0.000), students (*p* = 0.001), workers (*p* = 0.000) and retirees (*p* = 0.000). Figure 1 presents the differences in evolution over time, and comparisons between the professional statuses. The Kruskal-Wallis analyses show a significant difference concerning SSPA from family (*p* = 0.002). There are significant differences between students and workers (*p* = 0.008) and between students and retirees (*p* = 0.003).

**Table 2:** Descriptive statistics and evolutions over time.

	**Entire sample**					**Students**					**Workers**					**Retirees**				
	***N***	**Mean**	**SD**	***Z***	***p*-value**	***N***	**Mean**	**SD**	***Z***	***p*-value**	***N***	**Mean**	**SD**	***Z***	***p*-value**	***N***	**Mean**	**SD**	***Z***	***p*-value**
PA prior to lockdown	262	10.53	9.84	−1.239	0.215	38	10.22	8.56	−1.586	0.113	188	10.21	9.92	−0,49	0.624	36	12.50	10.66	−0,607	0.544
PA during lockdown	262	9.91	9.53			38	8.75	7.29			188	9.8	9.83			36	11.46	10.07		
Evolution of PA	262	−0.62	7.63	/	/	38	−1.47	6.21	/	/	188	−0.37	8.02	/	/	36	−1.04	6.92	/	/
Sedentariness prior to lockdown	262	34.81	25.78	−6.384	0.000	38	42.42	24.77	−1.423	0.155	188	35.29	25.93	−5,458	0.000	36	24.24	23.10	−3,875	0.000
Sedentariness during lockdown	262	40.31	27.54			38	45.13	26.11			188	41.24	28.17			36	30.31	23.76		
Evolution of sedentariness	262	5.50	13.77	/	/	38	2.71	15.38	/	/	188	5.95	14.20	/	/	36	6.07	8.76	/	/
SSPA from family prior to lockdown	272	1.04	0.63	−1.962	0.050	38	1.04	0.63	−2.191	0.028	195	1.04	0.63	−2,355	0.019	39	1.06	0.63	−2,219	0.026
SSPA from family during lockdown	272	1.00	0.68			38	1.22	0.58			195	0.98	0.69			39	0.90	0.71		
Evolution of SSPA from family	272	−0.04	0.49	/	/	38	0.17	0.49	/	/	195	−0.06	0.49	/	/	39	−0.15	0.43	/	/
SSPA from friends prior to lockdown	272	1.39	1.01	−10.129	0.000	38	1.64	1.02	−3,39	0.001	195	1.38	1.03	−8,69	0.000	39	1.16	0.88	−3,811	0.000
SSPA from friends during lockdown	272	0.71	0.81			38	0.97	1.01			195	0.68	0.80			39	0.56	0.59		
Evolution of SSPA from friends	272	−0.69	0.93	/	/	38	−0.67	1.02	/	/	195	−0.70	0.94	/	/	39	−0.59	0.83	/	/

### Bivariate Correlations

Bivariate correlations were examined for PA, sedentariness and SSPA variables according to professional statuses (see Tables 3–5). For each professional status, PA during lockdown is positively correlated with one or more PA' and SSPA from friends' variables, and negatively correlated with sedentariness during lockdown only on the part of students. Sedentariness during lockdown is positively correlated with sedentariness prior to lockdown for each professional status, negatively correlated with PA during lockdown for students, negatively correlated with one or more SSPA from family variables for students and workers, and positively correlated with SSPA from family prior to lockdown for retirees.

**Table 3:** Bivariate Correlations for Students (*n* = 38).

	**1**	**2**	**3**	**4**	**5**	**6**	**7**	**8**	**9**	**10**	**11**	**12**	**13**
1. Stage of change	–	0.55^**^	0.17	−0.27	−0.8	−0.08	0.00	0.22	0.04	−0.23	0.29	0.05	−0.17
2. PA prior to lockdown		–	0.31	−0.69^**^	−0.32^*^	−0.15	0.24	0.32	0.28	−0.10	0.30	0.24	0.00
3. PA during lockdown			–	0.38^*^	−0.20	−0.32^*^	−0.24	0.09	0.19	0.02	0.35^*^	0.51^**^	0.07
4. Evolution of PA				–	0.06	−0.21	−0.46^**^	−0.27	−0.17	0.13	0.08	0.15	−0.00
5. Sedentariness prior to lockdown					–	0.81^**^	−0.08	−0.03	−0.23	−0.23	−0.09	−0.33^*^	−0.21
6. Sedentariness during lockdown						–	0.46^**^	0.07	−0.21	−0.33^*^	−0.05	−0.17	−0.11
7. Evolution of sedentariness							–	0.09	−0.07	−0.22	0.06	0.17	0.13
8. SSPA from family prior to lockdown								–	0.66^**^	−0.51^**^	0.39^*^	0.15	−0.23
9. SSPA from family during lockdown									–	0.25	3.26	0.16	−0.15
10. Evolution of SSPA from family										–	−0.23	−0.06	0.19
11. SSPA from friends prior to lockdown											–	0.56^**^	−0.52^**^
12. SSPA from friends during lockdown												–	0.29
13. Evolution of SSPA from friends													–

* *P < 0.05*.

** *P <0.01*.

**Table 4:** Bivariate Correlations for Workers (*n* = 195).

	**1**	**2**	**3**	**4**	**5**	**6**	**7**	**8**	**9**	**10**	**11**	**12**	**13**
1. Stage of change	–	0.39^**^	0.28^**^	−0.01	−0.01	−0.03	−0.04	0.02	0.02	0.02	0.26^**^	0.15^*^	−0.17^*^
2. PA prior to lockdown		–	0.58^**^	−0.41^**^	0.08	0.15^*^	0.18^*^	−0.03	−0.04	−0.04	0.28^**^	0.13	−0.16^*^
3. PA during lockdown			–	0.41^**^	−0.01	−0.01	−0.14	0.10	0.09	−0.01	0.15^*^	0.11	−0.09
4. Evolution of PA				–	−0.11	−0.29^**^	−0.42^**^	0.14	0.13	−0.00	−0.06	0.03	0.06
5. Sedentariness prior to lockdown					–	0.88^**^	−0.11	−0.21^**^	−0.16^*^	0.02	−0.00	−0.18^*^	−0.10
6. Sedentariness during lockdown						–	−0.34^**^	−0.24^**^	−0.19^**^	0.01	0.03	−0.14	−0.11
7. Evolution of sedentariness							–	−0.11	−0.14	−0.08	0.03	0.04	−0.01
8. SSPA from family prior to lockdown								–	0.73^**^	−0.23^**^	0.25^**^	0.25^**^	−0.11
9. SSPA from family during lockdown									–	0.42^**^	0.19^**^	0.20^**^	−0.06
10. Evolution of SSPA from family										–	−0.06	−0.02	0.08
11. SSPA from friends prior to lockdown											–	0.49^**^	−0.65^**^
12. SSPA from friends during lockdown												–	0.25^**^
13. Evolution of SSPA from friends													–

* *P < 0.05*.

** *P < 0.01*.

**Table 5:** Bivariate Correlations for Retirees (*n* = 39).

	**1**	**2**	**3**	**4**	**5**	**6**	**7**	**8**	**9**	**10**	**11**	**12**	**13**
1. Stage of change	–	0.28	0.06	−0.24	−0.14	0.02	0.51^**^	0.16	0.07	−0.18	0.28	0.06	−0.22
2. PA prior to lockdown		–	0.79^**^	−0.23	0.31	0.31	0.12	0.10	−0.06	−0.28	−0.03	−0.25	−0.13
3. PA during lockdown			–	0.37^*^	0.23	0.09	−0.24	0.03	0.04	−0.02	0.02	−0.26	−0.21
4. Evolution of PA				–	−0.09	−0.21	−0.40^*^	−0.19	0.11	0.42^*^	−0.00	−0.06	−0.03
5. Sedentariness prior to lockdown					–	0.89^**^	0.10	0.37^*^	0.30	−0.08	0.05	0.02	−0.01
6. Sedentariness during lockdown						–	0.41^*^	0.37^*^	0.33	−0.02	−0.01	0.09	0.10
7. Evolution of sedentariness							–	0.09	0.09	0.03	0.08	0.25	0.12
8. SSPA from family prior to lockdown								–	0.82^**^	−0.16	0.49^**^	0.46^**^	−0.16
9. SSPA from family during lockdown									–	0.41^*^	0.41^**^	0.64^**^	0.06
10. Evolution of SSPA from family										–	−0.07	0.37^*^	0.36^*^
11. SSPA from friends prior to lockdown											–	0.41^*^	−0.74^**^
12. SSPA from friends during lockdown												–	0.26
13. Evolution of SSPA from friends													–

* *P < 0.05*.

** *P < 0.01*.

### Perception of COVID-19 Lockdown on Physical Activity

In chi-square analyses, the frequencies of perception of the influence of lockdown are significantly different depending on professional status (χ^2^ = 10.69, *p* = 0.030). As shown in Table 6, the proportion of students and workers who perceived lockdown as having a positive effect on their PA practice is higher than expected, while the proportion in terms of retirees is lower.

**Table 6:** Frequencies of experience of lockdown by professional status.

**Professional status**		**-**	**/**	**+**	**Total**
Students	Observed	12	7	19	38
	Expected	15.2	9.1	13.7	
	Chi-square	0.67	0.48	2.05	
Workers	Observed	77	45	73	195
	Expected	78.1	46.6	70.3	
	Chi-square	0.01	0.05	0.1	
Retirees	Observed	20	13	6	39
	Expected	15.6	9.3	14.1	
	Chi-square	1.24	1.47	4.65	
	Total	109	65	98	272

*Experience of lockdown: - indicates perception of a negative influence of lockdown on PA; / indicates no perception of influence; + indicates perception of a positive influence*.

### General Linear Models

In the general linear models, PA and sedentariness during lockdown were predicted by PA and sedentariness prior to lockdown, the evolution of SSPA from family and from friends, the SOC, and gender, together explaining, respectively, 59 and 57% of variance with regard to students (see Table 7), 45 and 75% of variance with regard to workers (see Table 8), and 77 and 91% of variance with regard to retirees (see Table 9). Among students, PA prior to lockdown (*F* = 45.460, *p* = 0.000) and SOC (*F* = 3.980, *p* = 0.012) are significantly associated with PA during lockdown; sedentariness prior to lockdown (*F* = 28.371, *p* = 0.000) is significantly associated with sedentariness during lockdown. Among workers, PA prior to lockdown (*F* = 134.396, *p* = 0.000) is significantly associated with PA during lockdown; PA prior to lockdown (*F* = 6.030, *p* = 0.015) and sedentariness prior to lockdown (*F* = 506.542, *p* = 0.000) are significantly associated with sedentariness during lockdown. Among retirees, PA prior to lockdown (*F* = 87.205, *p* = 0.000), the evolution of SSPA from family (*F* = 12.862, *p* = 0.001) and the evolution of SSPA from friends (*F* = 4.540, *p* = 0.043) are significantly associated with PA during lockdown; sedentariness prior to lockdown (*F* = 264.333, *p* = 0.000), the evolution of SSPA from friends (*F* = 4.663, *p* = 0.040) and the SOC (*F* = 3.394, *p* = 0.033) are significantly associated with sedentariness during lockdown.

**Table 7:** General linear model for students (*n* = 37).

	**PA during lockdown**			**Sedentariness during lockdown**		
	**Coefficient (B)**	**95% CI**		**Coefficient (B)**	**95% CI**	
		**Lower CI**	**Upper CI**		**Lower CI**	**Upper CI**
Constant	−2.145	−9.132	4.842	14.725	−7.975	37.426
PA prior to lockdown	0.740^**^	0.514	0.967	0.015	−0.720	0.749
Sedentariness prior to lockdown	0.025	−0.061	0.111	0.724^**^	0.444	1.004
Evolution of SSPA from family	1.662	−2.060	5.385	−9.284	−21.379	2.811
Evolution of SSPA from friends	1.178	−0.730	3.086	−0.771	−6.970	5.427
Precontemplation stage	−1.613	−7.052	3.825	11.068	−6.601	28.738
Contemplation stage	3.631	−4.426	11.687	4.834	−21.341	31.008
Preparation stage	11.272^*^	0.091	22.452	−8.548	−44.874	27.777
Action stage	11.758^**^	4.205	19.310	−17.425	−41.963	7.113
Maintaining stage	0			0		
Gender (female)	2.675	−2.068	7.418	−1.343	−16.752	14.066
Gender (male)	0			0		
Precontemplation stage X Female	0			0		
Contemplation stage X Female	−4.080	−14.940	6.781	9.023	−26.262	44.307
Contemplation stage X Male	0			0		
Preparation stage X Female	−9.032	−22.976	4.912	1.697	−43.606	46.999
Preparation stage X Male	0			0		
Action stage X Female	0			0		
Maintaining stage X Female	0			0		
Maintaining stage X Male	0			0		
R Squared (Adjusted R Squared)	0.717 (0.593)			0.703 (0.572)		

* *P < 0.05*,

** *P < 0.01*.

**Table 8:** General linear model for workers (*n* = 187).

	**PA during lockdown**			**Sedentariness during lockdown**		
	**Coefficient (B)**	**95% CI**		**Coefficient (B)**	**95% CI**	
		**Lower CI**	**Upper CI**		**Lower CI**	**Upper CI**
Constant	6.606^**^	3.293	9.918	5.138	−1.208	11.484
PA prior to lockdown	0.656^**^	0.544	0.768	0.266^*^	0.052	0.480
Sedentariness prior to lockdown	−0.023	−0.065	0.020	0.921^**^	0.840	1.001
Evolution of SSPA from family	−1.298	−3.531	0.934	0.652	−3.625	4.928
Evolution of SSPA from friends	0.535	−0.659	1.730	−0.488	−2.777	1.800
Precontemplation stage	2.668	−5.140	10.476	1.281	−13.676	16.238
Contemplation stage	−0.863	−15.590	13.863	−8.616	−36.826	19.594
Preparation stage	−1.587	−16.420	13.247	−7.295	−35.710	21.120
Action stage	−1.411	−11.970	9.149	6.037	−14.191	26.265
Maintaining stage	0			0		
Gender (female)	−2.249	−5.221	0.723	−0.553	−6.247	5.141
Gender (male)	0			0		
Precontemplation stage X Female	−4.326	−13.072	4.420	7.790	−8.964	24.544
Precontemplation stage X Male	0			0		
Contemplation stage X Female	−0.939	−16.378	14.500	9.038	−20.537	38.613
Contemplation stage X Male	0			0		
Preparation stage X Female	1.019	−14.208	16.246	3.967	−25.202	33.137
Preparation stage X Male	0			0		
Action stage X Female	−2.411	−13.646	8.825	−0.818	−22.341	20.704
Action stage X Male	0			0		
Maintaining stage X Female	0			0		
Maintaining stage X Male	0			0		
R Squared (Adjusted R Squared)	0.487 (0.448)			0.771 (0.754)		

* *P < 0.05*,

** *P < 0.01*.

**Table 9:** General linear model for retirees (*n* = 36).

	**PA during lockdown**			**Sedentariness during lockdown**		
	**Coefficient (B)**	**95% CI**		**Coefficient (B)**	**95% CI**	
		**Lower CI**	**Upper CI**		**Lower CI**	**Upper CI**
Constant	3.322	−2.345	8.989	5.945	−2.352	14.241
PA prior to lockdown	0.823^**^	0.642	1.004	0.029	−0.236	0.294
Sedentariness prior to lockdown	0.033	−0.055	0.120	1.014^**^	0.886	1.142
Evolution of SSPA from family	8.016^**^	3.422	12.610	0.785	−5.941	7.511
Evolution of SSPA from friends	−2.423^*^	−4.760	−0.086	3.595^*^	0.173	7.017
Precontemplation stage	−3.008	−11.070	5.054	−7.633	−19.436	4.169
Contemplation stage	5.793	−2.708	14.294	−14.307^*^	−26.752	−1.862
Preparation stage	15.334^**^	4.866	25.802	−14.283	−29.607	1.041
Maintaining stage	0			0		
Gender (female)	−6.032^**^	−11.050	−1.013	7.365^*^	0.017	14.712
Gender (male)	0			0		
Precontemplation stage X Female	7.608	−1.623	16.839	−3.500	−17.014	10.013
Precontemplation stage X Male	0			0		
Contemplation stage X Female	0			0		
Preparation stage X Female	0			0		
Maintaining stage X Female	0			0		
Maintaining stage X Male	0			0		
R Squared (Adjusted R Squared)	0.827 (0.767)			0.933 (0.910)		

* *P < 0.05*,

** *P < 0.01*.

## Discussion

In order to deal with the spread of COVID-19, the Belgian government opted for lockdown measures whereby schools were closed, homeworking became the new standard whenever possible, and social relations were greatly restricted. Sports centers, gyms and other indoor sports facilities were closed. Citizens were encouraged to engage in PA behaviors in order to maintain well-being during this major life-change event. More specifically, citizens were encouraged to engage in PA at home, or outdoors, alone, with members of the same household, or with a single friend. The results of the study highlight that the whole population did not share the same perception of the influence of COVID-19 lockdown on the practice of PA. Indeed, the findings suggest that workers, and especially students, are more likely to feel the influence of lockdown as positive in terms of their active behavior, unlike retirees who are more likely to feel it more negatively.

Beyond the perceptions of the influence of lockdown, it is interesting to examine the evolutions of PA and sedentary behaviors over time, between prior to and during lockdown, in order to better determine the impact of this event. The current study found no significant difference in the amount of PA, in terms of the total sample or by professional status. Although there was no significant difference in the amount of sedentariness on the part of students, the results of the study showed significant increases in sedentariness behavior among the total sample, workers and retirees. These findings corroborate those of Ammar et al. (2020) who reported a worldwide negative effect of COVID-19 lockdown on PA intensity levels, as well as an increase in time spent sitting per day. The results of the study by Tison et al. (2020) also showed a worldwide decrease in active behavior. Indeed, the authors reported a decrease in step counts in the period after COVID-19 was declared a global pandemic. However, the findings of the study partly differ from those of Constandt et al. (2020) who showed increased frequencies of both PA and sedentary behaviors among Belgian Flemish adults. In their study, the increase in the frequency of PA was particularly observed among people who were low in terms of being active prior to the lockdown. In our study, the sample of physically active people appears to be over-represented compared to the low active individuals, which may partially explain the difference in the evolution of PA behavior. A study carried out by López-Bueno et al. (2020) on Spanish adults provided an optimistic view of the influence of lockdown on health behaviors and the capacity of individuals to adapt to a life-change event. Indeed, although there was a decrease in the number of people reaching the PA recommendations during the 1st week of confinement, this quota increased progressively until it reached a higher level than before lockdown. However, it is also reported that screen time, which can be considered sedentary, increased during confinement and is maintained over time.

The findings of the study highlight the significant influence of past behaviors on current behaviors in the face of a life-change event that changed social relationships and opportunities. Indeed, the amount of PA practice and the amount of sedentary time prior to lockdown are, respectively, positively associated with the amounts of PA practice and sedentary times during lockdown. Moreover, these are the components that best predict active and sedentary behaviors, respectively, during this life-change event. These findings are consistent with the literature, which highlighted the importance of past behavior on current behavior. Indeed, studies showed that past PA behavior was a significant predictor of students' current practice when entering University life (Wing Kwan et al., 2009; Crozier et al., 2015). The literature and the results of this study support the importance of promoting health behaviors and countering sedentary behaviors during normal living, in order to maintain a healthy lifestyle in the face of a life-change event.

Although past behaviors seem to be the most important elements in the maintenance of health behaviors, some psychosocial determinants, such as SSPA, also appear to be important. Due to the measures related to the spread of COVID-19, restrictions on social contacts result in less SSPA from friends throughout the entire population. However, the findings of this study highlighted the associations between PA and SSPA from friends during lockdown on the part of students. This suggests that, as PA was allowed with one friend, students feel the need to take advantage of this opportunity to practice their PA with a friend. Another interesting finding is that the evolution of SSPA from family among students is significantly different from that among workers and retirees. The results of the study show an increase of SSPA from family among students, while they show a decrease among workers and retirees. The increase among students can be understood by the place of residence during their studies. Indeed, in Belgium, students generally live on University campuses. During the lockdown, most students returned to their families. Therefore, this potential source of support was much more available than in normal student life. The difference of evolutions of SSPA from family in terms of professional status might be a possible explanation for the difference of evolutions of sedentariness according to professional status. Indeed, the results of the study show no difference in the evolution of sedentariness among students while workers and retirees present a significant increase in sedentary behaviors. Furthermore, among retirees, SSPA from family and from friends participate in an understanding of PA practice during lockdown, and only SSPA from family participates in the outcomes with regard to sedentary behavior. These findings suggest that the support of individuals is particularly important among retirees. A previous systematic review of older adults highlighted that SSPA from family was an important factor with regard to being physically active (Lindsay Smith et al., 2017). Due to lockdown, the isolation of older people from their networks, and thus from their sources of SSPA, might therefore have a greater impact on this population group than on students or workers. In a critical commentaries issue related to COVID-19, Son et al. (2021) point out that older adults tend to be homebound, and needed alternative exercise and social opportunities to maintain their health during the COVID-19 lockdown. These authors suggested that leisure professionals can promote PA and social well-being in older people by increasing opportunities at home, including additional online leisure services, volunteering opportunities and social interactions.

### Limitations and Strengths

This study presents certain limitations. Firstly, this study includes a selection bias. Indeed, the sample was at convenience and not representative of the general population. It involves a sample of 272 individuals, in which physically active people is over-represented. In addition, the numbers of individuals in the students and retirees' subgroups are very small, this makes it difficult to draw conclusions. Future research could consider a larger sample of the population, one that is representative of the population with regard to different professional status and PA levels of individuals. Secondly, the measures prior to and during lockdown were self-reported and carried out at the same and single time. Despite the desire to carry out the survey very quickly at the beginning of the COVID-19 lockdown, the distance from habits prior to lockdown may be a bias of recall in terms of measurement. Thirdly, this study presents a confounding bias. Indeed, some demographic variables that could influence the data could have been considered in the statistical analyses, such as gender. In future research, confounding variables should be an integral part of the statistical analyses. Fourthly, the objective of the study was to investigate the possible link between SSPA and the subjects' practice of PA during the COVID-19 lockdown in Belgium. However, the SSES (Sallis et al., 1987) only gives overall indices of SSPA from family and friends. Future research could investigate the different forms of SSPA (informational, emotional, instrumental, co-participation and modeling) through the use of specific questionnaires, in order to observe the changes in forms of SSPA caused by a major life-change event. In addition, qualitative research could be of interest to gain a more in-depth understanding of the changes in health behaviors during such as drastic event.

Despite the above limitations, this study contains some strengths. The primary strength is that the authors do not only seek to observe activity and sedentary behaviors during lockdown, but also to observe changes compared to normal life. This was possible thanks to the quick diffusion of the survey at the beginning of the lockdown. Indeed, the intention was to observe the “acute” effect of this life-change event disrupting usual life. Moreover, the quick diffusion made it easier for participants to recall their pre-lockdown habits. The second strength of this study is that it seeks to understand these changes by relating the behaviors to some psychosocial elements such as SSPA. Finally, given the differences in social relationships and behaviors during normal life between groups of individuals, this study seeks to differentiate such groups in order to better understand the specific needs of each and best tailor subsequent recommendations.

## Conclusions

Promoting active behaviors is important in order to maintain physical and psychological well-being during a major life-change event such as a lockdown. However, recommendations should also address the need to counter sedentary behaviors that cause many health issues. Indeed, although physical activity has not changed significantly, this study shows an increase in sedentary behaviors on the part of all adults, with the exception of students, taken separately. To this end, promoting multi-person or group activities is a relevant means of increasing and maintaining active behaviors, and limiting sedentary behaviors. Indeed, the findings suggest that the support of other individuals could be useful for certain population groups such as retirees. Furthermore, given the importance of the levels of health behaviors preceding a major life-change event such as a lockdown, there is a need to also promote these health behaviors during normal life in order for the population to remain active throughout their lifespan.

## Data Availability Statement

The raw data supporting the conclusions of this article will be made available by the authors, without undue reservation.

## Ethics Statement

Ethical review and approval was not required for the study on human participants in accordance with the local legislation and institutional requirements. Written informed consent for participation was not required for this study in accordance with the national legislation and the institutional requirements.

## Author Contributions

CD, FD, and PV conceived the study. All authors were involved in analyzing of data, interpreting of results and revising the manuscript, and approve the final version of the paper.

## Conflict of Interest

The authors declare that the research was conducted in the absence of any commercial or financial relationships that could be construed as a potential conflict of interest.

## Publisher's Note

All claims expressed in this article are solely those of the authors and do not necessarily represent those of their affiliated organizations, or those of the publisher, the editors and the reviewers. Any product that may be evaluated in this article, or claim that may be made by its manufacturer, is not guaranteed or endorsed by the publisher.
